# FDA-Approved Drug Repurposing as p53 Mutants Rescue Candidates Using Structure-Based Virtual Screening and Molecular Simulations

**DOI:** 10.3390/ijms27156677

**Published:** 2026-07-27

**Authors:** Mena Abdelsayed, Yassir Boulaamane

**Affiliations:** 1 Lankenau Institute for Medical Research, Philadelphia, PA 19096, USA; 2 Laboratory of Innovative Technologies, National School of Applied Sciences of Tangier, Abdelmalek Essaadi University, Tetouan 93000, Morocco

**Keywords:** p53 mutant, drug repurposing, molecular dynamics simulations, virtual screening, structural stabilization, allosteric modulation

## Abstract

The restoration of mutant p53 stability is a highly sought-after strategy in targeted cancer therapy. This study presents a structure-based virtual screening and molecular dynamics approach to nominate FDA-approved drugs as candidate stabilizers of mutant p53 for downstream experimental validation. A virtual screening library of FDA-approved compounds was docked against three representative p53 mutants (7DHY, 7DHZ, and 7V97) to evaluate their binding potential. The prioritized candidates demonstrated consistent, multi-conformer binding affinities. Protein–ligand interaction profiling revealed that the candidate DB09280 possesses a highly dense interaction network, particularly against the V272M and R249S variants. Residue-level analysis of the G245S structural mutant showed that DB09280 uniquely engages His19, a crucial residue for zinc coordination, and forms stabilizing contacts with adjacent flexible loop residues, including ASN35 and PRO32. Subsequent 500 ns molecular dynamics simulations were consistent with DB09280 acting as a putative conformational clamp on the timescale sampled. The ligand-bound (holo) system exhibited substantially reduced global structural drift (RMSD) and attenuated local residue fluctuation (RMSF) within the core domain compared to the highly unstable apo state. Principal component analysis further indicated that DB09280 restricts the broad conformational sampling of the mutant into a stable, dominant basin within the sampled trajectory. Together, these computational findings nominate DB09280 as a promising candidate structural stabilizer of mutant p53 worthy of experimental follow-up. We emphasize that the in silico stabilization observed here is not equivalent to functional rescue of p53 transcriptional activity; biochemical, biophysical, and cell-based assays will be required to establish whether DB09280 restores wild-type-like DNA binding or tumor-suppressor function in mutant p53 contexts.

## 1. Introduction

The tumor suppressor protein p53 is a central guardian of genomic integrity, orchestrating cell-cycle arrest, apoptosis, and DNA damage responses in stressed cells. In human cancers, TP53 is the most frequently mutated gene, and these alterations often disable p53’s transcriptional activity, promote malignant progression, and correlate with poor clinical outcomes. Many oncogenic TP53 variants arise within the DNA-binding domain and can be broadly divided into “DNA-contact” mutations that directly impair sequence recognition and “structural” mutations that destabilize the loop–sheet–helix motif and the underlying β-sandwich scaffold. The latter class, which includes cavity-creating substitutions such as G245S, R249S, and V272M, compromises zinc coordination and reduces thermodynamic stability, predisposing p53 to partial unfolding and aggregation [1,2,3].

Reactivating mutant p53 has therefore emerged as an attractive yet challenging therapeutic strategy. Several approaches have been pursued, including small molecules that restore native-like conformation of structural mutants, compounds that refold misfolded p53, and agents that target auxiliary regulators such as MDM2 to indirectly enhance p53 signaling. However, most candidate reactivators show a narrow mutation spectrum, limited potency, or suboptimal pharmacokinetic properties, which has hindered their broad clinical translation. Recent work has revealed a cryptic allosteric site within the DNA-binding domain that can be targeted by cysteine-reactive small molecules such as arsenic trioxide (ATO), which stabilizes the loop–sheet–helix motif and β-sandwich fold of multiple structural p53 mutants. These studies provide a mechanistic blueprint for allosteric rescue, but the repertoire of clinically actionable ligands that exploit this pocket remains sparse [3,4].

Drug repurposing offers a complementary route to expand the pool of p53-stabilizing compounds by leveraging the safety and pharmacology of existing FDA-approved agents. Structure-based virtual screening combined with molecular dynamics (MD) simulations is particularly well suited to this task, because it can account for the pronounced conformational plasticity of mutant p53 and evaluate ligand binding across multiple disease-relevant variants. In the context of the arsenic-defined allosteric pocket, such an integrative in silico pipeline enables the identification of molecules that not only bind with favorable affinity but also act as dynamic stabilizers that clamp flexible loops and reinforce zinc-associated structural motifs [5]. Complementary molecular dynamics studies of the broader p53 regulatory axis—including polarizable MD with QM/MM–GBSA dissection of MDM2/MDMX inhibitor binding [6] and Gaussian-accelerated MD analyses of peptide and non-peptide MDM2 inhibitors using deep-learning-assisted scoring [7]—reinforce the value of long-timescale MD-driven workflows for prioritizing modulators of p53-related targets.

**Figure 1:**
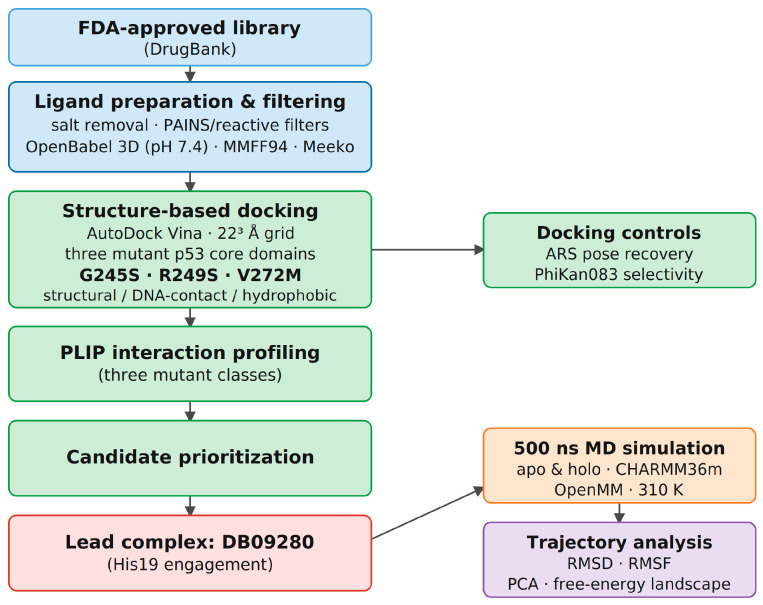
Overview of the computational workflow. FDA-approved compounds from DrugBank were filtered and prepared (salt removal, PAINS/reactive-substructure filtering, OpenBabel 3D generation at pH 7.4, MMFF94 optimization, Meeko PDBQT conversion) and docked against three mutant p53 structures (7DHY/G245S, 7DHZ/R249S, 7V97/V272M) with AutoDock Vina; poses were profiled with PLIP, and candidates were ranked (Table 1). The lead candidate DB09280 was advanced to 500 ns molecular dynamics of the apo and holo systems, followed by RMSD, RMSF, PCA and free-energy-landscape analysis. Positive (arsenic pose-recovery) and negative (PhiKan083 selectivity) docking controls validated the protocol.

In this study, we employed a structure-based virtual screening workflow to interrogate a curated library of FDA-approved drugs against three representative mutant p53 DNA-binding domains—G245S, R249S, and V272M—whose crystal structures capture distinct structural, DNA-contact, and hydrophobic perturbations. Top-ranked candidates were subjected to detailed protein–ligand interaction profiling at the arsenic-defined allosteric site, followed by long-timescale MD simulations to assess their ability to stabilize the mutant core domain. Our results highlight the compound DB09280 as a computational candidate stabilizer of mutant p53 that engages key residues involved in zinc coordination and loop flexibility and appears, on the simulation timescales studied here, to act as a putative conformational clamp on the G245S structural mutant. By integrating virtual screening, interaction fingerprinting, and MD-based conformational analysis, this work provides a hypothesis-generating computational framework for prioritizing FDA-approved scaffolds for downstream experimental validation as candidate mutant p53 stabilizers. An overview of the complete pipeline is shown in Figure 1.

**Table 1:** Candidate prioritization for the ten FDA-approved rescue candidates. Docking scores (kcal/mol) against the three mutant p53 structures 7DHY (G245S), 7DHZ (R249S) and 7V97 (V272M), their mean, the total PLIP interaction counts in each structure, and engagement of the rescue-relevant residue His19 (His20 in 7V97). DB09280 (bold) shows the highest interaction density in every structure and is the only candidate engaging His19 across all three, despite ranking fifth by mean docking score.

Candidate	7DHY (G245S)	7DHZ (R249S)	7V97 (V272M)	Mean	PLIP Contacts (G245S/R249S/V272M)	His19
DB11363	−8.26	−7.87	−7.64	−7.92	5/4/4	—
DB00762	−7.78	−7.74	−8.07	−7.86	8/6/8	—
DB06589	−7.88	−7.62	−7.62	−7.71	7/9/8	—
DB15133	−7.75	−7.59	−7.56	−7.64	8/6/5	—
**DB09280**	**−7.78**	**−7.37**	**−7.52**	**−7.56**	**9/10/11**	**All three**
DB08896	−7.24	−6.92	−8.06	−7.41	6/8/5	R249S
DB00619	−7.47	−7.45	−7.80	−7.58	6/7/7	—
DB15233	−7.88	−7.24	−7.32	−7.48	4/4/3	—
DB04868	−7.68	−6.80	−7.47	−7.32	6/6/5	—
DB11799	−7.20	−7.29	−7.95	−7.48	3/3/3	—

## 2. Results and Discussion

### 2.1. Structure-Based Virtual Screening Results

Molecular docking was performed against three mutant p53 crystal structures (7DHY, 7DHZ, and 7V97) to evaluate the binding potential of the top ten rescue candidates. Overall, the compounds exhibited consistently favorable binding affinities across all conformations, with docking scores ranging from −6.8 to −8.3 kcal/mol, indicating stable interactions within the mutant p53 binding pocket. Across the full screened FDA-approved library, docking scores spanned −8.26 to −1.28 kcal/mol, placing these ten candidates within the top ~1% of the distribution. Because the ~1.5 kcal/mol spread among the top candidates lies within the intrinsic uncertainty of the AutoDock Vina scoring function, the docking stage was used as an enrichment and prioritization filter rather than as a quantitative affinity ranking. Among the screened molecules, DB11363 emerged as the strongest binder to the 7DHY structure (−8.3 kcal/mol), while DB00762 and DB08896 showed the highest affinity toward 7V97 (both −8.1 kcal/mol). Several candidates, including DB06589, DB15133, and DB09280, demonstrated reproducible binding across all three structures, suggesting robustness against p53 conformational variability. Notably, DB00619 displayed balanced affinities across the panel, with improved binding to 7V97 (−7.8 kcal/mol), whereas DB15233 and DB04868 showed slightly reduced interaction energies for 7DHZ, reflecting possible structural sensitivity of this mutant form. Interestingly, DB08896 exhibited a marked increase in affinity toward 7V97 despite weaker binding to 7DHZ, highlighting potential mutation-specific stabilization effects. These results indicate that multiple candidates possess consistent multi-conformer binding capability, a critical property for rescuing structurally heterogeneous mutant p53. On this basis, the ten compounds exhibiting consistent multi-conformer binding were advanced to protein–ligand interaction profiling (Table 1); from this shortlist, DB09280 was subsequently elevated for detailed dynamic characterization on the basis of its interaction-fingerprint density and its unique engagement of His19 (Section 2.2), rather than on docking rank alone.

**Figure 2:**
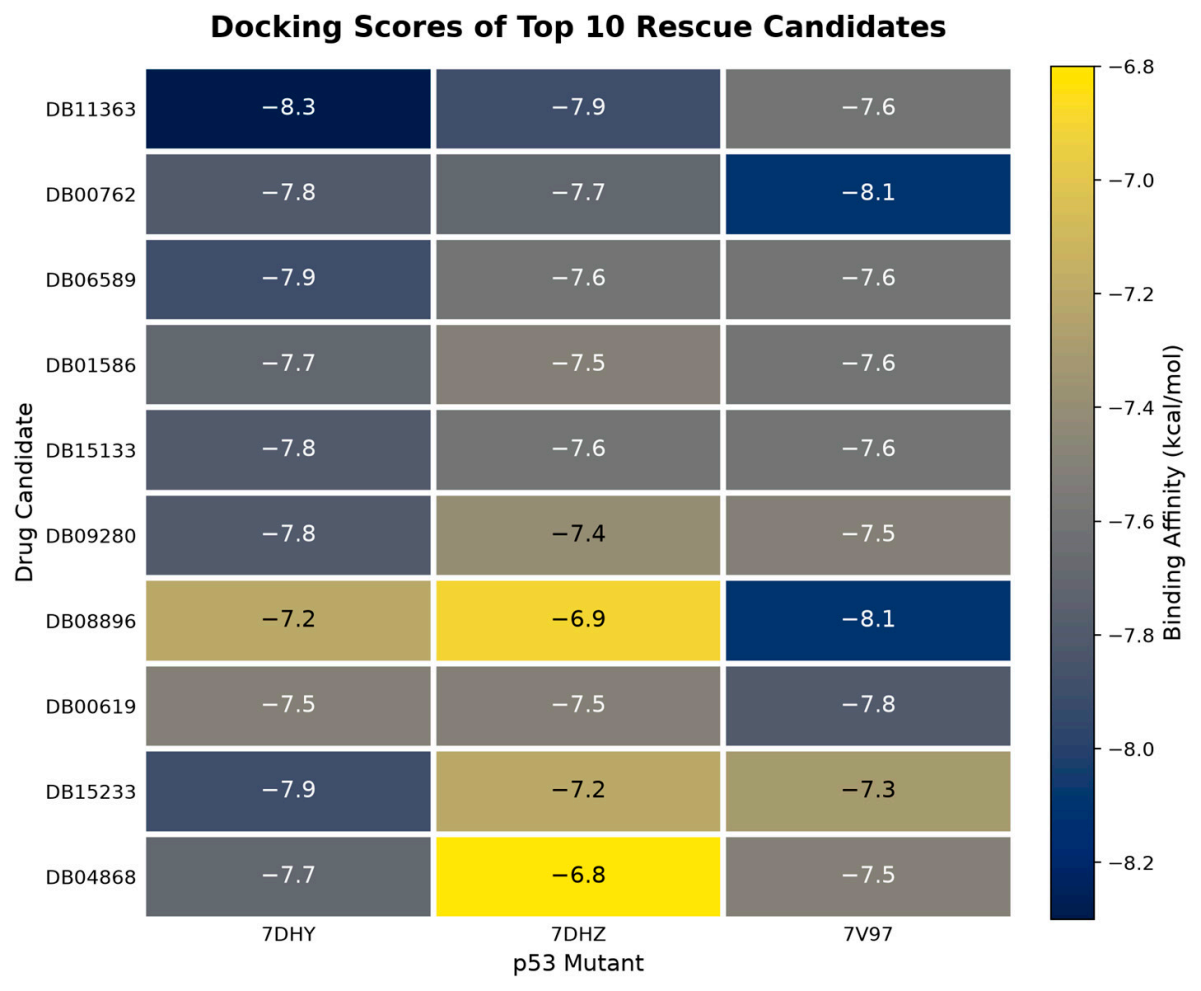
Docking scores (kcal/mol) of the top ten rescue candidates against three mutant p53 crystal structures (7DHY, 7DHZ, and 7V97). Lower (more-negative) values indicate stronger predicted binding affinity. The heatmap highlights consistency and mutation-specific differences in binding profiles across conformational variants.

The observed binding consistency across multiple mutant p53 conformations is particularly relevant given the structural plasticity associated with oncogenic p53 variants. Compounds exhibiting stable affinity across 7DHY, 7DHZ, and 7V97 are more likely to tolerate mutation-induced pocket rearrangements and may function as structural stabilizers rather than mutation-specific binders. The enhanced affinity of DB11363 and DB00762 suggests stronger stabilization potential, whereas DB08896’s mutation-dependent variability may indicate selective conformational preference. These findings support a mechanism in which small molecules bind to destabilized regions of mutant p53, potentially restoring partial structural integrity and functional activity (Figure 2). The docking protocol was validated using two controls (Section 3.3): the crystallographic arsenic species was recovered within the search grid in all three structures, confirming that the grid captures the experimentally defined rescue site, whereas PhiKan083 [8]—a stabilizer of the spatially distinct Y220C cleft that is absent in these mutants—docked approximately 2–3 kcal/mol more weakly and away from the arsenic site, demonstrating site selectivity.

### 2.2. Protein–Ligand Interactions Analysis

**Figure 3:**
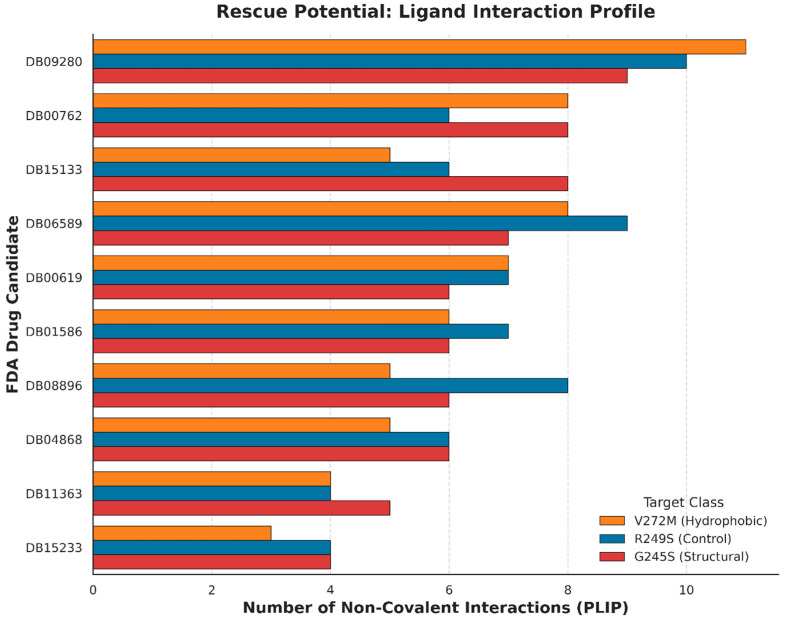
Non-covalent interaction profiles of prioritized FDA-approved candidates across three mutant p53 classes (V272M, R249S, and G245S), calculated using PLIP. Bars represent the total number of stabilizing interactions, including hydrogen bonds, hydrophobic contacts, salt bridges, and π-stacking interactions.

To complement docking scores and evaluate the structural stabilization potential of selected candidates, protein–ligand interaction profiles were analyzed using PLIP across three representative mutant p53 classes: V272M (hydrophobic), R249S (DNA-contact), and G245S (structural). Distinct interaction patterns were observed among the compounds, reflecting mutation-dependent binding behavior. DB09280 exhibited the highest overall interaction density, particularly against V272M (11 interactions) and R249S (10 interactions), suggesting strong anchoring within the destabilized pocket. DB06589 and DB00762 also demonstrated elevated interaction counts across multiple variants, indicating consistent engagement with key binding residues. Several compounds, including DB15133 and DB08896, showed preferential interaction with specific mutants, with DB08896 displaying enhanced binding to R249S despite reduced contacts with V272M. In contrast, DB11363 and DB15233 exhibited comparatively weaker interaction profiles, consistent with their moderate docking performance. Importantly, multiple candidates displayed balanced interaction distributions across all three mutant classes, highlighting their potential to act as general p53 stabilizers rather than mutation-specific ligands (Figure 3).

To gain mechanistic insight into ligand-mediated stabilization of mutant p53, residue-level interaction fingerprints were generated for the G245S structural mutant. This analysis revealed recurrent engagement of key residues within the DNA-binding core domain, including THR6, ARG14, LEU15, PHE17, TYR30, PRO32, ASN35, LYS68, LEU56, ASN72, and GLU75.

**Figure 4:**
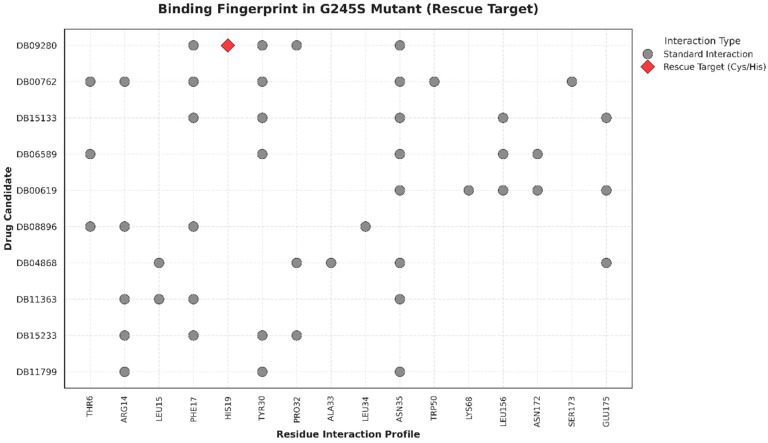
Binding fingerprint of prioritized rescue candidates within the G245S mutant p53 core domain. Dots indicate ligand–residue contacts identified by PLIP. Gray circles represent standard non-covalent interactions, while red diamonds highlight direct engagement with rescue-relevant residues (Cys/His). Residues are shown along the x-axis and compounds along the y-axis.

DB09280 displayed the most extensive interaction network, contacting multiple hydrophobic and polar residues and uniquely engaging His19, a residue implicated in zinc coordination and structural stabilization. This interaction is highlighted as a potential rescue hotspot, suggesting a direct contribution to restoring local folding integrity. Several candidates, including DB06589, DB00619, and DB00762, showed consistent contacts with ASN35, LEU56, and ASN72, residues located within flexible regions surrounding the mutation site. Such multi-point anchoring may counteract the destabilizing effect of G245S by reinforcing local secondary structure elements. In contrast, DB11363 and DB15233 exhibited fewer residue contacts, consistent with their weaker interaction density observed in earlier PLIP analyses (Figure 4).

The binding fingerprints indicate that effective rescue candidates preferentially interact with residues surrounding the destabilized β-sandwich of mutant p53. In particular, engagement of His19 by DB09280 suggests a potential mechanism involving reinforcement of zinc-associated structural motifs, a known strategy for restoring mutant p53 stability. The presence of dense interaction clusters around ASN35, LEU56, and ASN72 further supports a model in which ligands stabilize flexible loops adjacent to the mutation site, thereby promoting partial refolding toward a native-like conformation. These residue-level interactions provide molecular evidence that top candidates act as structural stabilizers rather than simple pocket binders, supporting their advancement to molecular dynamics simulations for validation of long-timescale rescue effects.

**Figure 5:**
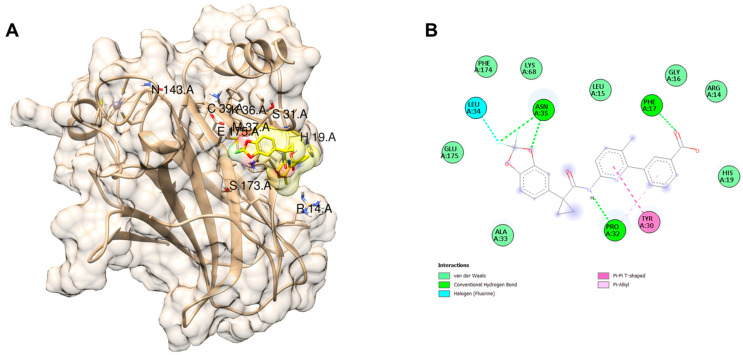
Structural binding mode of DB09280 within the G245S mutant p53 core domain. (**A**) Three-dimensional representation of the ligand (shown in yellow color) bound within the p53 cavity, shown in surface and cartoon view, highlighting key interacting residues surrounding the mutation site. (**B**) Two-dimensional interaction map illustrating hydrogen bonds, van der Waals contacts, and π-mediated interactions between DB09280 and critical p53 residues.

To visualize the molecular basis of mutant p53 rescue, the binding pose of the top-ranked candidate DB09280 was examined in detail within the G245S structure. As shown in Figure 5A, DB09280 occupies a hydrophobic pocket adjacent to the mutation site, engaging residues distributed across flexible loop regions and β-sheet elements of the DNA-binding domain. The ligand establishes a dense interaction network involving ASN35, PRO32, TYR30, LEU34, PHE17, and HIS19, forming a stabilizing scaffold around the destabilized core (Figure 5B). Multiple hydrogen bonds anchor the ligand to ASN35 and PRO32, while π-π and π–alkyl interactions with TYR30 and surrounding hydrophobic residues further reinforce binding.

The structural binding mode of DB09280 reveals a multi-point stabilization strategy, in which the ligand bridges flexible loops and β-sheet elements surrounding the G245S mutation. Rather than acting as a simple affinity-driven binder, DB09280 appears to function as a conformational clamp, reinforcing local structural elements and potentially restoring native-like folding. The interaction with HIS19 is particularly noteworthy, as perturbation of zinc coordination is a hallmark of structural p53 mutants. By engaging this residue, DB09280 may partially reconstitute the metal-supported architecture of the DNA-binding domain, providing a plausible molecular basis for rescue activity.

### 2.3. Molecular Dynamics Trajectory Analysis

#### 2.3.1. Root Mean Square Deviation

To evaluate the structural impact of ligand binding on mutant p53 stability, 500 ns molecular dynamics simulations were performed for both apo and holo systems.

**Figure 6:**
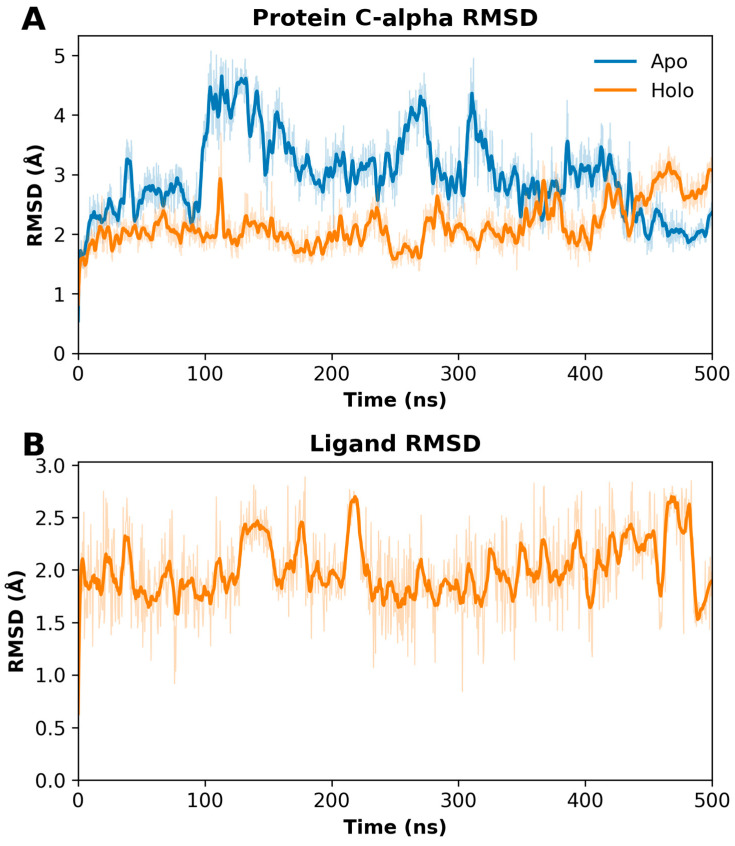
Molecular dynamics analysis of mutant p53 in apo and holo forms over 500 ns. (**A**) Cα RMSD of the protein backbone for apo (blue) and ligand-bound (holo, orange) systems. (**B**) Ligand RMSD relative to the initial docked conformation during the simulation.

The Cα RMSD profile (Figure 6A) reveals larger and more-sustained structural fluctuations in the apo system than in the holo complex. Over the equilibrated interval (100–500 ns), the apo protein exhibits a mean Cα RMSD of 3.09 ± 0.69 Å, compared with 2.18 ± 0.39 Å for the holo complex, with correspondingly smaller block-averaged uncertainties (0.15 versus 0.08 Å). Across consecutive 50 ns windows the holo system consistently maintains a lower average RMSD and a smaller fluctuation amplitude, indicating ligand-induced conformational stabilization; neither system, however, reaches a strictly flat plateau, consistent with the intrinsic conformational lability of these destabilized structural mutants. Although both systems display intrinsic flexibility, consistent with the destabilized nature of structural p53 mutants, the ligand-bound complex demonstrates reduced amplitude and shorter-lived excursions from equilibrium, indicating partial structural constraint. Ligand RMSD analysis (Figure 6B) further supports stable complex formation. The ligand maintains deviations primarily within 1.5–2.5 Å throughout the 500 ns trajectory, without evidence of dissociation or major reorientation. Transient increases are observed but rapidly re-equilibrate, indicating persistent pocket occupancy and sustained interactions. The MD results reinforce the docking and interaction fingerprint analyses by demonstrating dynamic stabilization of mutant p53 upon ligand binding. While the apo structure exhibits large conformational excursions characteristic of destabilized mutants, the holo system shows comparatively restrained fluctuations, particularly during the later stages of the simulation. The stable ligand RMSD confirms that the compound remains firmly anchored within the binding pocket, enabling continuous multi-point interactions that likely reinforce local secondary structure elements. Importantly, the reduction in large-scale structural drift in the holo system supports a rescue-like mechanism driven by conformational constraint rather than global rigidification. This behaviour is consistent with small-molecule-mediated stabilization of partially unfolded p53 variants.

#### 2.3.2. Root Mean Square Fluctuation

**Figure 7:**
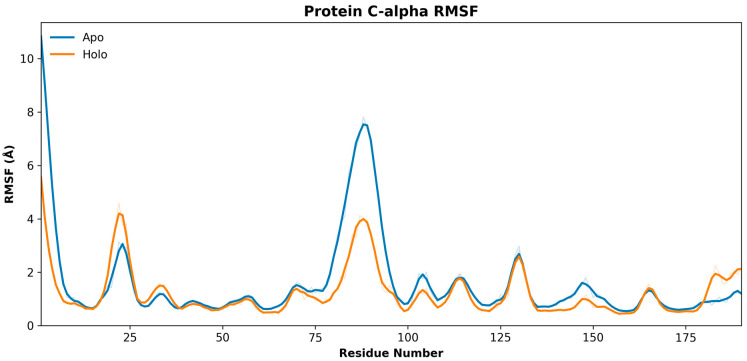
Cα RMSF profiles of mutant p53 in apo (blue) and ligand-bound (holo, orange) states over 500 ns MD simulation. Lower RMSF values indicate reduced residue-level flexibility upon ligand binding.

Residue-wise root mean square fluctuation (RMSF) analysis was performed to assess local backbone dynamics of mutant p53 in the presence and absence of the bound ligand. Overall, the holo system exhibits reduced fluctuations across multiple regions of the protein compared to the apo form (Figure 7), indicating ligand-mediated dampening of local flexibility. The most pronounced differences are observed in the central core domain, particularly around residues ~80–100, where the apo structure displays elevated mobility reaching ~7 Å, whereas the holo complex shows substantially attenuated fluctuations (~4 Å). This region encompasses structural elements proximal to the G245S mutation and is critical for maintaining the integrity of the DNA-binding domain. Additional stabilization is observed in loop regions near residues ~20–30 and ~120–140, consistent with earlier interaction fingerprint analyses showing ligand engagement with residues in these flexible segments. Although terminal regions retain higher mobility, as expected for solvent-exposed tails, the core domain demonstrates clear rigidity upon ligand binding. The reduction in residue-level fluctuations within the p53 core domain suggests that ligand binding restricts excessive conformational freedom induced by the G245S mutation. By stabilizing flexible loops and adjacent secondary structure elements, the ligand likely promotes a more compact and native-like fold. This localized rigidity complements the global RMSD stabilization and supports a rescue mechanism driven by reinforcement of structurally compromised regions rather than uniform protein rigidification.

### 2.4. Principal Component Analysis

**Figure 8:**
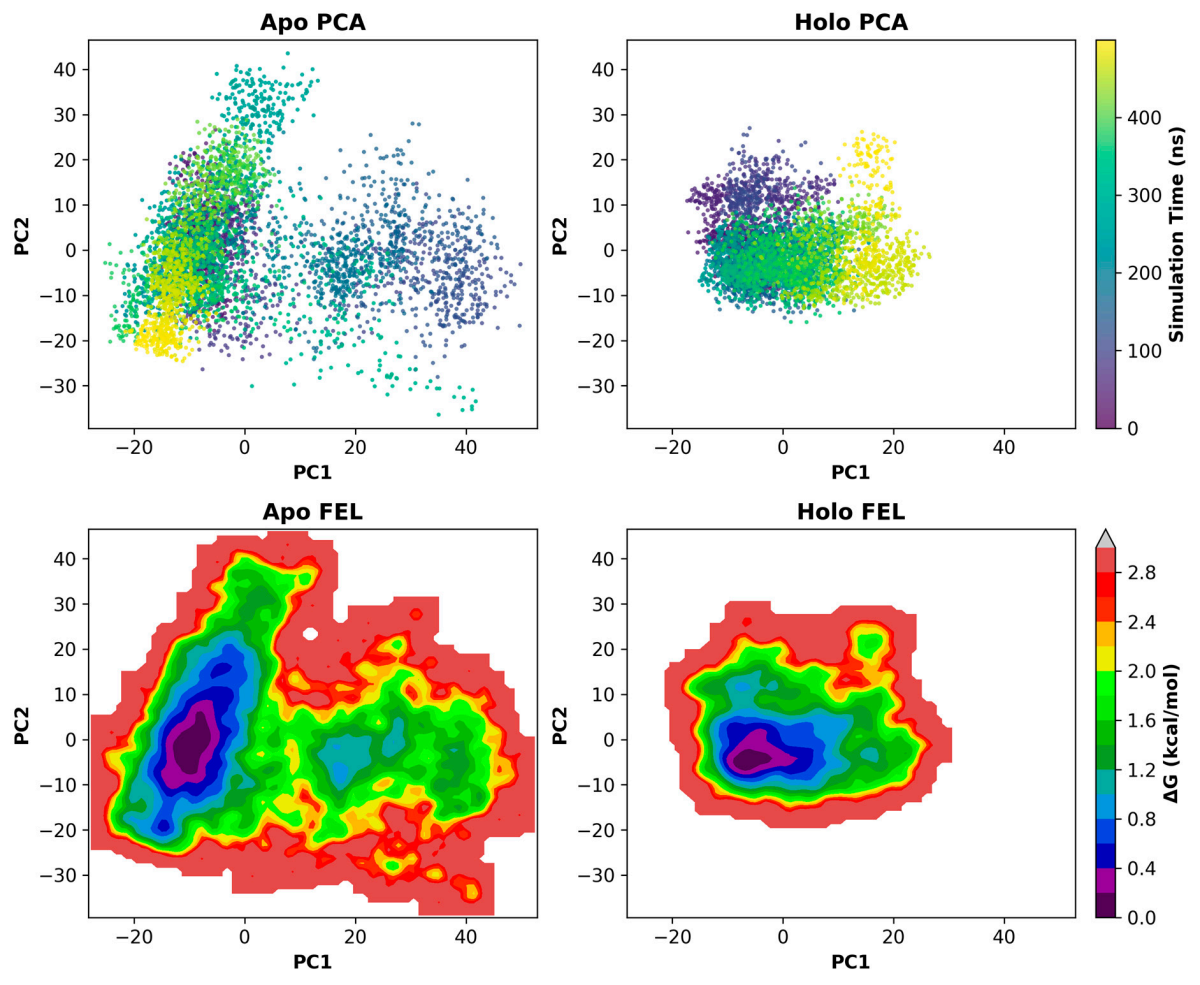
Principal component analysis (PCA) of mutant p53 dynamics over 500 ns MD simulations in apo (**left**) and ligand-bound (holo, **right**) states. Upper panels show projections along the first two principal components colored by simulation time; lower panels show the corresponding free-energy landscapes obtained by Boltzmann inversion of the PC1–PC2 histogram. Projections along the first two principal components (PC1 and PC2) illustrate the conformational space sampled during the trajectories, colored by simulation time.

Principal component analysis was performed to characterize large-scale collective motions of mutant p53 in apo and holo states. Projection of the trajectories onto the first two principal components reveals markedly different conformational sampling between the two systems (Figure 8).

The apo protein explores a broad and highly dispersed conformational space, indicative of pronounced structural heterogeneity and dynamic instability. Multiple clusters are observed, reflecting frequent transitions between distinct conformational substates throughout the simulation.

In contrast, the holo complex exhibits a more compact and structured distribution along PC1 and PC2, with trajectory points concentrating within fewer dominant regions. This reduced dispersion suggests ligand-induced restriction of global motions and stabilization of preferred conformational states. Time-colored projections further indicate progressive convergence toward a stable basin in the holo system, whereas the apo form continues to sample diverse conformations over the entire trajectory. The PCA results are consistent with the interpretation that ligand binding reshapes the conformational sampling of mutant p53 by narrowing accessible structural states and promoting population of a dominant basin. Quantitatively, the trace of the Cα covariance matrix—a single-number measure of overall flexibility—decreased from 1000 Å^2^ in the apo system to 440 Å^2^ in the holo complex, and the sampled 2σ PC1–PC2 area contracted from 2492 to 774 (a 3.2-fold reduction); PC1 and PC2 captured 25% and 16% of the variance for the apo system versus 17% and 12% for the holo, and cosine-content values (0.19 and 0.003 for apo PC1/PC2; 0.40 and 0.06 for holo) indicate that the dominant collective motions are physical rather than random-walk artifacts. To convert these projections into an interpretable energy surface, a free-energy landscape (FEL) was constructed by Boltzmann inversion of the two-dimensional PC1–PC2 probability histogram, ΔG = −k_BT·ln(P/P_max) (Figure 8, lower panels): the apo protein populates a broad, rugged surface with a principal basin and several shallow secondary minima, whereas the holo complex collapses to a single, compact, well-defined basin, directly visualizing the ligand-induced contraction of the accessible conformational space. As only a single replica was simulated per system, this landscape is interpreted as descriptive of the sampled trajectory rather than as a fully converged thermodynamic surface. Methodologies that build quantitative free-energy or kinetic descriptions on top of PCA—for example Markov state modeling and communication-pathway analyses [9] or Gaussian-accelerated MD trajectory-image transformations coupled to deep learning [10]—represent natural extensions of the present work and would allow unambiguous quantification of basin depths and inter-state transition rates. This behavior is compatible with small-molecule-mediated conformational stabilization, where flexible and partially unfolded regions become constrained into energetically favorable arrangements. Together with RMSD and RMSF analyses, these findings support a mechanism in which the ligand acts as a dynamic stabilizer, suppressing aberrant motions associated with the G245S mutation and facilitating adoption of more native-like conformations.

## 3. Materials and Methods

### 3.1. p53 Mutant Preparation

Three relevant p53 mutants namely, G245S, R249S, and V272M (PDB IDs: 7DHY, 7DHZ, and 7V97, respectively) were retrieved from the RCSB Protein Data Bank (PDB) [11]. Structures were selected based on the availability of high-resolution transmembrane domains, the presence of resolved or inferred fenestration pathways, and their relevance to clinically validated channelopathies (Table 2). Prior to molecular docking, all protein structures were subjected to standardized preprocessing and optimization. Protonation states of titratable residues were assigned using the H++ server (http://newbiophysics.cs.vt.edu/H++/ (accessed on 2 June 2026)) at physiological pH [12]. Crystallographic water molecules were removed. Atom types and Gasteiger partial charges were then assigned using ForliLab’s Meeko package, and the prepared structures were converted to PDBQT format for subsequent docking simulations [13].

### 3.2. Ligand Library Preparation

FDA-approved compounds were extracted from DrugBank to construct the virtual screening library. Ligand structures were initially provided in SDF format and subjected to salt removal using RDKit’s SaltRemover module [14]. Reactive electrophilic substructures were filtered based on predefined SMARTS patterns, followed by elimination of pan-assay interference compounds (PAINS) using RDKit’s FilterCatalog to reduce false-positive risk. For each retained molecule, explicit hydrogens were added and a single three-dimensional conformer was generated with OpenBabel (--gen3d) with protonation assigned at pH 7.4. Geometry optimization was then performed with the MMFF94 force field (obminimize, up to 1000 iterations) to obtain energetically reasonable ligand conformations prior to docking [12,15] The optimized ligands were then processed with Meeko (mk_prepare_ligand.py), which assigned Gasteiger partial charges, detected rotatable bonds, preserved stereochemistry, and converted structures into PDBQT format compatible with AutoDock Vina v1.2.7 [16,17].

### 3.3. Molecular Docking

Molecular docking was performed using AutoDock Vina to evaluate binding of FDA-approved compounds to the allosteric pocket of mutant p53 corresponding to the arsenic-binding rescue site. The docking region was defined based on the experimentally characterized allosteric cavity, previously reported as a targetable rescue hotspot. The center of the docking grid was determined in UCSF Chimera using the “measure center” command applied to the arsenic-binding site, yielding coordinates of x = −34.188, y = 3.782, and z = −7.713 [14]. A cubic grid box of 22 × 22 × 22 Å was then centered on these coordinates to fully encompass the allosteric pocket and surrounding flexible regions of the DNA-binding domain, ensuring adequate sampling of ligand conformations. Prepared ligands in PDBQT format were docked against the prepared mutant p53 structures using AutoDock Vina with an exhaustiveness of 8, generating up to nine binding poses per ligand (default energy range). For each ligand, the top-ranked pose based on predicted binding affinity was retained for downstream analysis. Docking results were subsequently processed to extract best-scoring complexes, which were converted to PDB format for protein–ligand interaction profiling and molecular dynamics simulations [18,19]. To validate the docking protocol, two controls were carried out with the identical grid. As a positive (pose-recovery) control, the crystallographic arsenic species present in 7DHY, 7DHZ and 7V97 was used: after superposition of the three receptors onto a common frame, the arsenic atom lay 5.1–5.6 Å from the grid center, with the coordinating Cys124/Cys135/Cys141 cluster 4.8–5.6 Å away—well within the 22 Å box—confirming recovery of the only experimentally defined binding mode at this site. Because arsenic binds as a trivalent metalloid coordinated to three cysteine thiolates, for which empirical scoring functions are not parameterized, it was used as a geometric pocket-location control rather than as a scored ligand. As a negative (selectivity) control, PhiKan083 [20], a stabilizer of the Y220C-specific surface cleft that is spatially distinct from and absent in the G245S/R249S/V272M mutants, was docked into the same grid; it scored −5.3 to −5.8 kcal/mol (2–3 kcal/mol weaker than the prioritized candidates) and localized approximately 14 Å from the arsenic site. Software versions used were as follows: AutoDock Vina 1.2.0 (Scripps Research, La Jolla, CA, USA), Open Babel 3.1.1, RDKit 2024.09.2, Meeko 0.7.1, OpenMM 8.3.1 (Stanford University, Stanford, CA, USA), PLIP 3.0.0 (TU Dresden, Dresden, Germany), and MDAnalysis 2.10.0.

### 3.4. Molecular Dynamics Simulations

The top-ranked compounds identified from molecular docking were selected for molecular dynamics simulations to evaluate the stability and dynamic behavior of their complexes with mutant p53. Protein–ligand systems were constructed using the CHARMM-GUI web interface, applying the CHARMM36m force field for the protein and associated parameters for the ligands [17,18]. The complex was embedded in a cubic TIP3P water box with a minimum padding of 10 Å from the solute to the box boundary. Sodium and chloride ions were added to neutralize the systems and adjust ionic strength to 0.15 M. Periodic boundary conditions were applied, and long-range electrostatics were treated using the Particle Mesh Ewald method with automatically generated FFT grid dimensions. Energy minimization was performed to remove unfavorable contacts, followed by stepwise equilibration under constant-volume (NVT) and constant-pressure (NPT) conditions at 310 K and 1 bar, respectively. Temperature regulation was achieved using a Langevin thermostat, while pressure coupling was maintained with a Monte Carlo barostat. The cubic simulation box measured 68 × 68 × 68 Å and comprised 29,221 atoms for the apo system (including 8721 TIP3P water molecules and 24 Na^+^/28 Cl^−^ ions) and 29,269 atoms for the holo system. Following energy minimization, the systems were equilibrated for 125 ps of NVT dynamics at 310 K under positional restraints (force constants of 1.0 and 0.1 kcal mol^−1^ A^−2^ applied to backbone and side-chain heavy atoms, respectively). Production simulations were subsequently carried out for 500 ns each using OpenMM with a 2 fs integration timestep, all bonds to hydrogen constrained, a Langevin thermostat (310 K, 1 ps^−1^ friction) and a Monte Carlo barostat (1 bar) [15]. Trajectory frames were saved every 0.1 ns (50,000 steps) for downstream analysis. Post-simulation analyses, including root mean square deviation (RMSD), root mean square fluctuation (RMSF), principal component analysis (PCA), and ligand stability assessment, were conducted using MDTraj and MDAnalysis, as implemented in the analysis notebook [19,20]. A single 500 ns production replica was performed for each system. We acknowledge that single-replica MD samples only a fraction of the accessible conformational landscape and that multiple independent replicas are generally preferred for rigorous statistical assessment of stabilization effects; the analyses presented here are therefore interpreted as descriptive of the trajectories sampled rather than as a definitive characterization of equilibrium thermodynamics. Convergence of the RMSD/RMSF baselines and of the dominant principal component basin during the latter portion of each trajectory was used as an internal consistency check on the sampled regime. We also note that AutoDock Vina docking scores are inherently approximate and are not intended as quantitative free energies; in this work they were used only as an initial enrichment filter, with downstream prioritization driven primarily by consistency of binding poses across mutants, interaction-fingerprint density, and MD-derived stability metrics.

## 4. Conclusions

The therapeutic reactivation of mutant p53 requires small molecules capable of counteracting mutation-induced thermodynamic instability. Through an integrated computational pipeline comprising molecular docking, interaction fingerprinting, and 500 ns molecular dynamics simulations, we evaluated FDA-approved drugs as prospective candidate stabilizers of mutant p53. Our virtual screening identified several compounds with robust, cross-mutant binding profiles, highlighting DB09280 as a standout candidate capable of tolerating structural plasticity. Structural interaction analyses revealed that DB09280 functions not merely as an affinity-driven pocket binder, but as a conformational clamp that reinforces local secondary structure elements. By strategically anchoring to flexible loops and directly engaging His19, a residue vital for zinc coordination, the ligand addresses the core structural deficits characteristic of the G245S mutation. Long-timescale dynamic analyses strongly corroborated this stabilization mechanism. The holo complex exhibited profound reductions in global RMSD and localized RMSF within the compromised central core domain. Furthermore, principal component analysis demonstrated that ligand binding effectively limits aberrant large-scale motions, forcing the destabilized mutant to converge into a stabilized, native-like conformational state. These in silico findings nominate DB09280 as a candidate structural stabilizer of mutant p53. This study provides a hypothesis-generating computational rationale for prioritizing DB09280 and structurally related FDA-approved scaffolds for evaluation in in vitro and in vivo models—including thermal-shift, DNA-binding, and cell-based reporter assays—that can directly probe whether the structural stabilization observed in silico translates into restoration of p53 tumor-suppressor function.

## 5. Limitations and Scope of the Present Study

Several important caveats temper the interpretation of the present results and define the scope to which our conclusions should be confined. First, our workflow is entirely computational. Structural stabilization of the mutant p53 core domain on the molecular-dynamics timescale, as inferred from RMSD, RMSF, and PCA analyses, is not equivalent to functional rescue of p53 tumor-suppressor activity. Restoration of wild-type-like DNA binding, transcriptional activation of canonical p53 target genes (e.g., CDKN1A/p21, MDM2, BAX, PUMA), induction of cell-cycle arrest or apoptosis in mutant p53 cancer cell lines, and demonstrable on-target engagement in cellular thermal-shift or related assays will all be required to establish whether DB09280 acts as a genuine functional reactivator rather than a non-specific structural binder. Second, AutoDock Vina docking scores were used as an enrichment filter rather than as quantitative free-energy estimates; their well-known limitations in flexible, partially destabilized systems such as mutant p53 mean that pose plausibility, multi-conformer consistency, and downstream MD-derived metrics—not docking energies per se—drove candidate prioritization. Third, the production simulations consisted of a single 500 ns replica per system. Single-replica MD samples only a portion of the accessible conformational landscape and is, in general, insufficient for rigorous statistical claims about stabilization or convergence; multiple independent replicas, longer aggregate sampling, and enhanced sampling approaches (e.g., Gaussian-accelerated MD, replica exchange, metadynamics) will be needed to confirm convergence and to construct quantitative free-energy landscapes from the principal component subspace. Fourth, the free-energy landscapes shown in the lower panels of Figure 8 were obtained by Boltzmann inversion of the two-dimensional PC1–PC2 probability histograms; because they derive from a single trajectory per system, they should be interpreted as descriptive of the sampled conformational space rather than as fully converged thermodynamic surfaces, and combining them with Markov-state models or related kinetic frameworks would be needed to quantify basin depths and inter-state transition rates rigorously. Fifth, the simulations do not explicitly model the catalytic Zn^2+^ rescue mechanisms involving zinc reloading or stabilization of the zinc-coordinating tetrad cannot, therefore, be ruled in or ruled out from the present data. As a specific methodological extension, we plan to apply QM/MM and QM/MM-derived end-point free-energy approaches (e.g., QM/MM-GBSA) to the DB09280–mutant p53 complexes in future work, in order to characterize the binding energetics and the electronic nature of their noncovalent interactions near the zinc-coordinating region more precisely than classical force fields allow. Finally, this study evaluated three structural variants (G245S, R249S, V272M); generalization to the full mutational spectrum of TP53—including hotspot DNA-contact mutants such as R175H and R273H—will require dedicated docking, MD, and experimental work. Taken together, the findings reported here should be regarded as hypothesis-generating computational evidence that prioritizes DB09280 and related FDA-approved scaffolds for systematic biochemical, biophysical, and cellular validation, rather than as a closed mechanistic case for functional p53 reactivation.

## Figures and Tables

**Table 2:** p53 Mutants Used for Virtual Screening and Molecular Dynamics.

p53 Variant	PDB ID	Mutation Class	Structural Consequence
G245S	7DHY	Structural	Destabilizes local secondary structure elements and perturbs critical zinc coordination loops.
R249S	7DHZ	DNA-contact	Disrupts direct interactions with consensus DNA sequences without completely unfolding the core.
V272M	7V97	Hydrophobic	Alters internal hydrophobic packing within the core beta-sandwich, inducing thermodynamic instability.

## Data Availability

The data supporting this study—docking configuration files, prepared input structures (PDBQT/PSF/PDB/CRD), OpenMM run scripts, and the analysis notebooks and scripts—are openly available on Zenodo at https://doi.org/10.5281/zenodo.21506800. Additional inquiries can be directed to the corresponding author.
